# Daratumumab ± pomalidomide maintenance after salvage autologous transplantation in relapsed multiple myeloma: a phase 2 study

**DOI:** 10.1038/s41408-026-01620-w

**Published:** 2026-09-26

**Authors:** Oren Pasvolsky, Bruno Almeida Costa, Yosra M. Aljawai, Krina K. Patel, Qaiser Bashir, Dawen Sui, Samer A. Srour, Mark R. Tanner, Uday R. Popat, Neeraj Y. Saini, Chitra Hosing, Jeremy L. Ramdial, Hans C. Lee, Gregory P. Kaufman, Sheeba K. Thomas, Donna M. Weber, Melody Becnel, Guilin Tang, Robert Z. Orlowski, Richard E. Champlin, Yago Nieto, Elizabeth J. Shpall, Muzaffar H. Qazilbash

**Affiliations:** 1 Department of Lymphoma & Myeloma, The University of Texas MD Anderson Cancer Center, Houston, TX USA; 2 Division of Cancer Medicine, The University of Texas MD Anderson Cancer Center, Houston, TX USA; 3 Department of Stem Cell Transplantation & Cellular Therapy, The University of Texas MD Anderson Cancer Center, Houston, TX USA; 4 Department of Biostatistics, The University of Texas MD Anderson Cancer Center, Houston, TX USA; 5 Myeloma Research Program, Sarah Cannon Research Institute, Nashville, TN USA; 6 Department of Hematopathology, The University of Texas MD Anderson Cancer Center, Houston, TX USA; 7 Department of Experimental Therapeutics, The University of Texas MD Anderson Cancer Center, Houston, TX USA

**Keywords:** Medical research, Myeloma

Dear Editor,

Salvage or delayed autologous stem cell transplantation (ASCT) has historically been an important treatment option for relapsed/refractory multiple myeloma (RRMM). However, its role has been challenged in recent years with the emergence of highly active T-cell–redirecting therapies, including bispecific antibodies (BsAbs) and chimeric antigen receptor (CAR) T-cell therapy [1]. Despite these advances, salvage ASCT remains a valuable alternative for inducing deep and durable remissions in well-selected patients, although relapse ultimately occurs in most cases [2].

Maintenance therapy following frontline ASCT has been standard practice for over a decade, supported by multiple randomized controlled trials (RCTs) demonstrating improved progression-free survival (PFS) with single-agent lenalidomide or combination approaches incorporating proteasome inhibitors or anti-CD38 monoclonal antibodies. Reflecting these data, current National Comprehensive Cancer Network guidelines list daratumumab (DARA) among recommended post-ASCT maintenance options and endorse a two-drug maintenance regimen (typically combining an anti-CD38 monoclonal antibody with lenalidomide) for patients with high-risk disease [1].

In contrast, prospective data on maintenance strategies after salvage or delayed ASCT remain scarce. Retrospective studies suggest that maintenance therapy may improve outcomes compared with observation in this setting, yet optimal regimens have not been clearly defined [3–5]. Given the well-demonstrated activity of DARA in RRMM and its emerging role in post-transplant maintenance for newly diagnosed disease, we performed a single-center phase 2 trial of DARA with or without pomalidomide (POM) as maintenance therapy following salvage or delayed ASCT in patients with RRMM.

This open-label, single-arm phase 2 study (ClinicalTrials.gov ID: NCT03622775) was conducted at The University of Texas MD Anderson Cancer Center to evaluate DARA-based maintenance after salvage or delayed ASCT for RRMM. Our protocol initially specified DARA monotherapy but was amended shortly after activation to allow the addition of POM because many transplant candidates were lenalidomide-refractory and/or anti-CD38-exposed. The study was approved by the institutional review board, and all participants provided written informed consent. All procedures were conducted in accordance with the Declaration of Helsinki and Good Clinical Practice guidelines.

Eligible patients were adults (≥18 years) with RRMM who achieved at least a partial response to their most recent therapy and underwent ASCT 60–180 days before enrollment. Additional eligibility criteria included Eastern Cooperative Oncology Group (ECOG) performance status ≤2 and adequate organ function. Key exclusion criteria were non-secretory disease, plasma cell leukemia, active central nervous system involvement, prior allogeneic transplantation, and known intolerance or refractoriness to either study drug. High-risk cytogenetic status was retrospectively classified per 2025 International Myeloma Society (IMS)–International Myeloma Working Group (IMWG) criteria using comprehensive cytogenetic data collected at baseline [6]; high tumor burden was defined as ≥50% bone marrow plasma cells, and true extramedullary disease as soft-tissue plasmacytomas noncontiguous with bone.

Maintenance therapy was administered in 28-day cycles. DARA (1800 mg subcutaneously) was given weekly during weeks 1–8, every 2 weeks during weeks 9–24, and every 4 weeks thereafter. POM (2 mg orally) was given on days 1–21 of each cycle in participants receiving combination therapy. The primary endpoint was PFS, defined as the time from maintenance initiation to disease progression or death. Secondary endpoints included overall survival (OS), hematologic response per IMWG criteria, measurable residual disease (MRD) negativity at 6 months (±3 months; 10^–5^ sensitivity level by next-generation flow cytometry performed per the EuroFlow consortium protocol), and safety [7].

The planned sample size was 56 patients to detect a 40% hazard reduction in PFS versus historical controls (two-sided *α* = 0.05, 90% power). Enrollment was terminated early because of slow accrual; reported analyses are thus descriptive, including frequencies (percentages) for categorical variables and medians (ranges) for continuous variables. Time-to-event outcomes were estimated using Kaplan–Meier methods, and median follow-up using the reverse Kaplan–Meier approach. Exploratory restricted mean survival time (RMST) was calculated as area under Kaplan–Meier curve from diagnosis to 10-year truncation point, with 95% confidence intervals (CIs) from percentile bootstrap resampling (5000 replicates) [8]. Statistical analyses were performed in SAS 9.4 (SAS Institute, Cary, NC) and plots were generated in RStudio 2022.12.0 (Posit PBC, Boston, MA).

Thirteen patients were enrolled between May 2019 and August 2022. Baseline characteristics are summarized in Table 1. At salvage/delayed ASCT, median age was 64 years (range, 43–76) and median time from diagnosis was 58.3 months (range, 7.6–132.1). Patients had received a median of 2 prior lines of therapy (range, 2–4). For the 6 previously transplanted participants (46%), median interval between first and salvage ASCT was 71.7 months (range, 53.2–125.8); the remaining 7 participants underwent their first (delayed) ASCT before study enrollment. High-risk cytogenetics were present in 5 patients (38%), high tumor burden in 8 (62%), and true extramedullary disease in 2 (15%).

**Table 1:** Baseline characteristics of the study population.

Characteristic^a^	*N* = 13
Age, years — median (range)	64 (43–76)
Time from diagnosis, months — median (range)	58.3 (7.6–132.1)
Female sex — no. (%)	6 (46%)
Race — no. (%)	
White	9 (69%)
Black	3 (23%)
Other	1 (8%)
Hispanic/Latino ethnicity — no. (%)	1 (8%)
Performance status — no. (%)	
ECOG 1	12 (92%)
ECOG 2	1 (8%)
eGFR per CKD-EPI equation — no. (%)	
≥ 60 mL/min/1.73 m^2^	9 (69%)
30-60 mL/min/1.73 m^2^	3 (23%)
< 30 mL/min/1.73 m^2^	1 (8%)
HCT–CI score — median (range)	4 (0–8)
HCT–CI score ≥ 3 — no. (%)	8 (62%)
Heavy chain isotype — no. (%)	
IgG	7 (54%)
IgA	5 (38%)
Light chain only	1 (8%)
Light chain type — no. (%)	
Kappa	11 (85%)
Lambda	2 (15%)
High-risk cytogenetics^b^ — no. (%)	5 (38%)
True extramedullary disease — no. (%)	2 (15%)
High tumor burden (≥50% BMPCs) — no. (%)	8 (62%)
Number of prior therapy lines — median (range)	2 (2-4)
Number of prior therapy lines — no. (%)	
Two	9 (69%)
Three	3 (23%)
Four	1 (8%)
Previous ASCT — no. (%)	6^c^ (46%)
Treatment refractoriness/exposure — no. (%)	
Lenalidomide refractory	9 (69%)
Double-class refractory^d^	6 (46%)
Pomalidomide exposed	5 (38%)
Triple-class exposed	6 (46%)
Penta-drug exposed^e^	2 (15%)
Reinduction regimen — no. (%)	
PI/IMID-based triplet	7^f^ (54%)
Daratumumab-based triplet	4^g^ (31%)
Elotuzumab-based triplet	2^h^ (15%)
Conditioning regimen — no. (%)	
MEL200	5 (38%)
MEL140	3 (23%)
BUMEL	4 (31%)
PANO-GEM-BUMEL	1 (8%)

*ASCT* autologous hematopoietic stem cell transplantation, *BMPCs* bone marrow plasma cells, *BUMEL* busulfan (pharmacokinetically-adjusted intravenous dose targeting a daily area under the curve of 4000–5000 µM·min) plus melphalan 100–140 mg/m², *CKD-EPI* Chronic Kidney Disease Epidemiology Collaboration, *ECOG* Eastern Cooperative Oncology Group, *eGFR* estimated glomerular filtration rate, *GEM* gemcitabine, HCT–CI hematopoietic cell transplantation–comorbidity index, *Ig* immunoglobulin, *IMID* immunomodulatory imide drug, *MEL140* melphalan 140 mg/m², *MEL200* melphalan 200 mg/m², *PANO* panobinostat, *PI* proteasome inhibitor.

^a^All characteristics were assessed at the time of salvage/delayed ASCT, except cytogenetic risk status, which was assigned retrospectively using baseline cytogenetic data.

^b^Defined by the presence of ≥1 of the following criteria: (1) del(17p) and/or *TP53* mutation; (2) *t*(4;14), *t*(14;16), or *t*(14;20) co-occurring with 1q gain/amplification and/or del(1p32); (3) monoallelic del(1p32) co-occurring with 1q gain/amplification, or biallelic del(1p32).

^c^Includes 1 patient who previously underwent tandem ASCT as frontline consolidative strategy.

^d^Failure to achieve ≥PR or disease progression within 60 days of last exposure to ≥1 IMID and ≥1 PI.

^e^Prior exposure to ≥2 IMIDs, ≥2 PIs, and ≥1 anti-CD38 monoclonal antibody.

^f^Includes carfilzomib/pomalidomide/dexamethasone (*n* = 3), bortezomib/lenalidomide/dexamethasone (*n* = 2), carfilzomib/lenalidomide/dexamethasone (*n* = 1), and ixazomib/lenalidomide/dexamethasone (*n* = 1).

^g^Includes daratumumab/lenalidomide/dexamethasone (*n* = 2), daratumumab/pomalidomide/dexamethasone (*n* = 1), and daratumumab/carfilzomib/dexamethasone (*n* = 1).

^h^Includes elotuzumab/pomalidomide/dexamethasone (*n* = 1) and elotuzumab/bortezomib/dexamethasone (*n* = 1).

At data cutoff (November 25, 2025), the median follow-up from maintenance initiation was 48.5 months (95% CI, 37.3–59.8). Study treatment began a median of 3.6 months (95% CI, 2.3–5.4) after ASCT, with 2 patients (15%) receiving DARA alone and 11 (85%) receiving the DARA-POM doublet. Median duration of maintenance therapy was 32.6 months. Overall, 6 participants had progression and 2 died, one from progressive disease and one from COVID-19.

Median PFS from maintenance initiation was 45.1 months (95% CI, 26.3–not reached [NR]; Fig. 1A) and median OS was NR (95% CI, 51.1–NR; Fig. 1B). Landmark analysis from salvage/delayed ASCT showed 4-year PFS of 62% (95% CI, 35–88) and 4-year OS of 100% (Table S1). Exploratory analysis of OS from initial diagnosis estimated a 10-year RMST of 117.9 months (95% CI, 112.8–120.0).

**Fig. 1:**
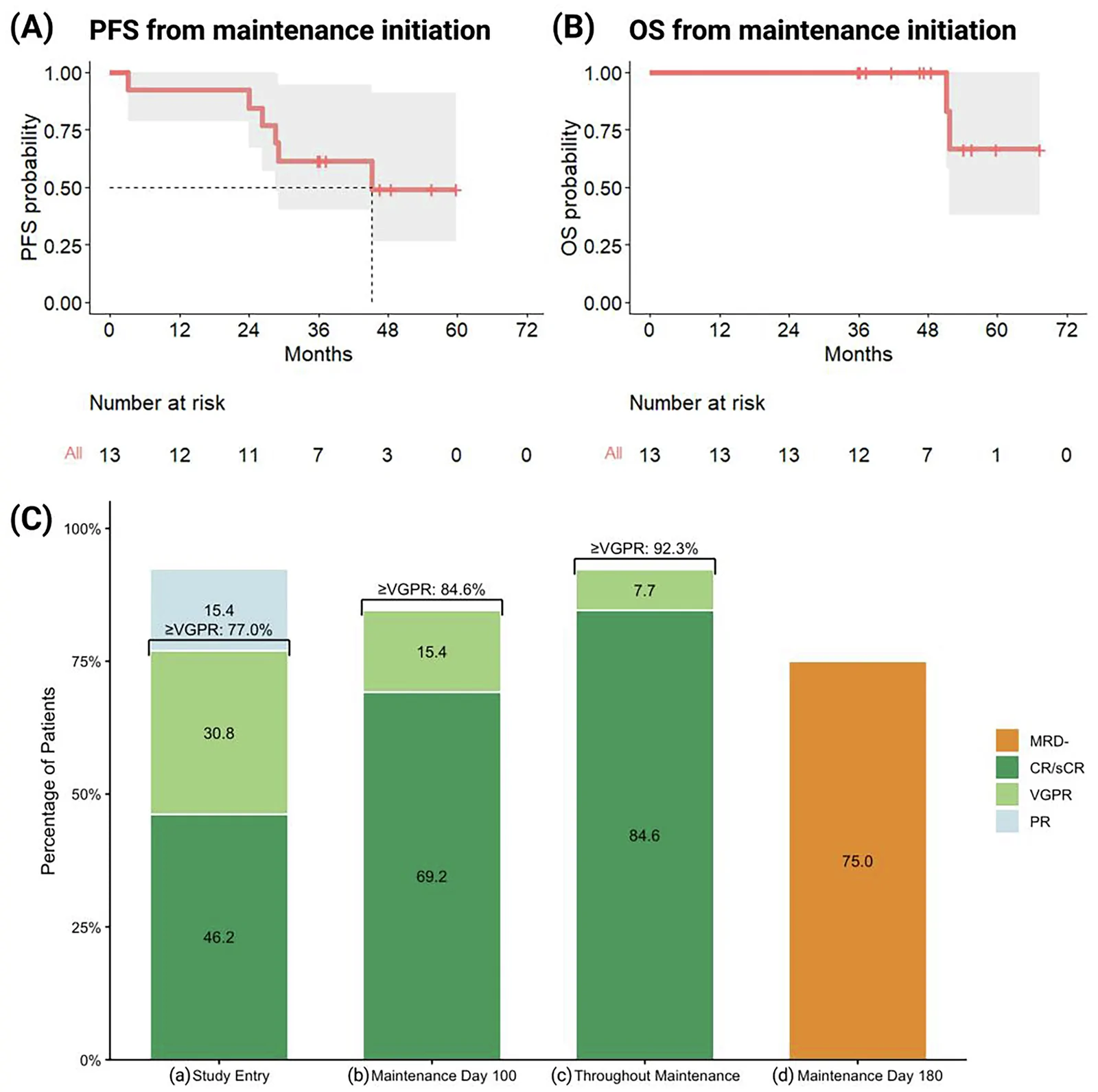
Efficacy outcomes in the study population. **A** Kaplan-Meier curve of PFS from maintenance initiation. **B** Kaplan-Meier curve of OS from maintenance initiation. **C** Longitudinal disease assessments, including (a) distribution of response categories at study entry (pre-maintenance), (b) distribution of response categories at day 100 post-maintenance initiation, (c) best hematological response achieved throughout the maintenance course, and (d) MRD− at day 180 post-maintenance initiation. Abbreviations: CR/sCR complete response or stringent complete response, MRD− measurable residual disease negativity rate (<10^–5^ level), OS overall survival, PFS progression-free survival, PR partial response, VGPR very good partial response.

There was a steady increase in the depth of response during maintenance (Fig. 1C). The proportion of patients in complete response (CR) or better increased from 46% at maintenance initiation to 85% at the final response evaluation. Among 7 patients entering maintenance with <CR, 5 (71%) ultimately achieved ≥CR. Among 8 patients with evaluable MRD data, 6 (75%) were MRD-negative at 6 months.

Table S2 summarizes adverse events observed during the study. The most common grade ≥3 toxicity was neutropenia (62%), while other cytopenias were grade 1–2 only. Grade ≥3 bacterial or viral infections were seen in 31% of patients. Non-hematologic toxicities were generally mild, with fatigue and diarrhea limited to grade 1–2 severity.

In this phase 2 study, DARA-based maintenance following salvage or delayed ASCT was feasible and associated with deepening responses over time in patients with RRMM. The ≥CR rate increased to 85% during maintenance, and MRD negativity (<10^–5^ level) was observed in 75% of evaluable patients. With a median follow-up approaching four years, median PFS reached 45.1 months among patients who had 2–4 prior treatment lines. Ten-year RMST from diagnosis was 9.8 years, consistent with the favorable long-term outcomes achievable in the current era of anti-myeloma therapy. Maintenance was reasonably well-tolerated, with reported adverse events aligning with the known toxicity profiles of each agent.

Although cross-study comparisons should be interpreted cautiously, these outcomes appear favorable relative to contemporary reports of salvage ASCT, where median PFS typically ranges from 17 to 31 months [2–5, 9–12]. Importantly, our analyses measured PFS from maintenance initiation rather than from ASCT, a landmark approach that differs from many such prior studies and, if anything, yields more conservative time-to-event estimates. Furthermore, our findings are consistent with evidence supporting DARA-based maintenance after frontline ASCT, including results from the CASSIOPEIA and AURIGA trials in newly diagnosed multiple myeloma [13, 14]. The present study notably extends this concept to the relapsed setting and suggests that DARA-based maintenance may represent a rational strategy after salvage ASCT. For instance, in a single-center retrospective cohort of post–second ASCT maintenance with bortezomib (*n* = 15) or lenalidomide (*n* = 23), median PFS was 15 and 25 months, respectively [3]. Similarly, among patients who underwent salvage ASCT followed by lenalidomide maintenance in the phase 3 ReLApsE trial (*N* = 102), median PFS was 23.0 months [12]. Another phase 3 RCT, the ACCoRD study (*N* = 103), reported a median PFS of 20.0 months in salvage ASCT recipients randomized to ixazomib-based consolidation followed by ixazomib maintenance [9].

Several limitations warrant acknowledgment. Early closure due to slow accrual resulted in a small sample size, limiting statistical precision and generalizability—constraints further amplified by the single-center, nonrandomized design. The protocol amendment introducing POM created treatment heterogeneity and precluded regimen-specific comparisons. The exploratory RMST analysis should also be interpreted with caution, as the limited number of deaths and total follow-up modestly shorter than the truncation horizon may affect estimate stability. Finally, MRD data were available for only a subset of patients, with a lack of serial testing at consistent longitudinal intervals precluding determination of sustained MRD negativity.

As the therapeutic landscape of RRMM continues to evolve rapidly with the expansion of CAR T-cell therapy and BsAbs into earlier treatment lines, the role of salvage ASCT may be increasingly questioned, though likely to maintain relevance for years to come, particularly in lower-resource settings [1]. Despite unprecedented response rates, CAR T-cell therapy has been associated with a 1-year non-relapse mortality of 9% versus 0% for salvage ASCT in a multicenter retrospective cohort [15]. Therefore, salvage ASCT followed by well-tolerated outpatient maintenance may remain a clinically meaningful strategy.

In conclusion, this single-center phase 2 trial supports DARA ± POM as a feasible and well-tolerated maintenance strategy following salvage or delayed ASCT, with the potential to yield deep responses and prolonged PFS in RRMM, while also reinforcing that salvage transplantation remains a viable therapeutic option for appropriately selected patients. These results, however, should be interpreted in the context of the small sample size resulting from early termination and the treatment heterogeneity following protocol amendment, both of which may limit the generalizability and strength of our findings.

## Supplementary information

Supplement

## Data Availability

De-identified individual participant data underlying our results may be made available upon reasonable request. All proposals should be directed to the corresponding author (mqazilba@mdanderson.org), requiring independent review by the institutional data access committee.
