# Supplementary material for: Daratumumab ± pomalidomide maintenance after salvage autologous transplantation in relapsed multiple myeloma: a phase 2 study
Source: Blood Cancer J. 2026 Sep 26;16(1):163. doi: 10.1038/s41408-026-01620-w (PMC13615963; doi:10.1038/s41408-026-01620-w)
Supplement: Supplementary file 1 [file 41408_2026_1620_MOESM1_ESM.docx]

**SUPPLEMENTARY MATERIAL**

**TABLE S1. Milestone survival rates:** Kaplan-Meier estimates of overall survival and progression-free survival at 12-month intervals from different time origins (study entry; salvage/delayed autologous stem cell transplantation; and daratumumab ± pomalidomide maintenance initiation).

| **Survival**  **Start Point** | **Time (mo)** | **Overall Survival** | | | **Progression-Free Survival** | | |
| --- | --- | --- | --- | --- | --- | --- | --- |
|  |  | **Events** | **N at risk** | **Rate (95% CI)** | **Events** | **N at risk** | **Rate (95% CI)** |
| **Date of study entry** | 0 | 0 | 13 | 1.00 (1.00; 1.00) | 0 | 13 | 1.00 (1.00; 1.00) |
|  | 12 | 0 | 13 | 1.00 (1.00; 1.00) | 1 | 12 | 0.92 (0.78; 1.07) |
|  | 24 | 0 | 13 | 1.00 (1.00; 1.00) | 1 | 12 | 0.92 (0.78; 1.07) |
|  | 36 | 0 | 13 | 1.00 (1.00; 1.00) | 5 | 8 | 0.62 (0.35; 0.88) |
|  | 48 | 0 | 7 | 1.00 (1.00; 1.00) | 6 | 3 | 0.49 (0.19; 0.79) |
|  | 60 | 2 | 1 | 0.67 (0.29; 1.04) | 6 | 0 | N/A |
| **Date of salvage/delayed transplantation** | 0 | 0 | 13 | 1.00 (1.00; 1.00) | 0 | 13 | 1.00 (1.00; 1.00) |
|  | 12 | 0 | 13 | 1.00 (1.00; 1.00) | 1 | 12 | 0.92 (0.78; 1.07) |
|  | 24 | 0 | 13 | 1.00 (1.00; 1.00) | 1 | 12 | 0.92 (0.78; 1.07) |
|  | 36 | 0 | 13 | 1.00 (1.00; 1.00) | 5 | 8 | 0.62 (0.35; 0.88) |
|  | 48 | 0 | 9 | 1.00 (1.00; 1.00) | 5 | 5 | 0.62 (0.35; 0.88) |
|  | 60 | 2 | 2 | 0.67 (0.29; 1.04) | 6 | 1 | 0.46 (0.13; 0.79) |
| **Date of maintenance initiation** | 0 | 0 | 13 | 1.00 (1.00; 1.00) | 0 | 13 | 1.00 (1.00; 1.00) |
|  | 12 | 0 | 13 | 1.00 (1.00; 1.00) | 1 | 12 | 0.92 (0.78; 1.07) |
|  | 24 | 0 | 13 | 1.00 (1.00; 1.00) | 2 | 11 | 0.85 (0.65; 1.04) |
|  | 36 | 0 | 12 | 1.00 (1.00; 1.00) | 5 | 7 | 0.62 (0.35; 0.88) |
|  | 48 | 0 | 7 | 1.00 (1.00; 1.00) | 6 | 3 | 0.49 (0.19; 0.79) |
|  | 60 | 2 | 1 | 0.67 (0.29; 1.04) | 6 | 0 | N/A |

**TABLE S2. Treatment-related adverse events (N=13).** Incidence presented as n (%); dashes indicate zero events. Toxicities are organized by clinical category and graded per NCI-CTCAE version 4.0 (only the maximum grade per event reported for each patient). Columns are arranged with the all-grade summary first, followed by individual grades in ascending order and the grade ≥3 composite; bold values highlight all-grade and grade ≥3 occurrences. No grade 5 events were observed.

| **Adverse event** | **All grades n (%)** | **Grade 1 n (%)** | **Grade 2 n (%)** | **Grade 3 n (%)** | **Grade 4 n (%)** | **Grade ≥3 n (%)** |
| --- | --- | --- | --- | --- | --- | --- |
| **Hematologic** |  |  |  |  |  |  |
| Neutropenia | **11 (85)** | 2 (15) | 1 (8) | 6 (46) | 2 (15) | **8 (62)** |
| Thrombocytopenia | **5 (38)** | 4 (31) | 1 (8) | — | — | **—** |
| Anemia | **3 (23)** | 2 (15) | 1 (8) | — | — | **—** |
| Leukopenia | **2 (15)** | 2 (15) | — | — | — | **—** |
| Lymphopenia | **2 (15)** | 1 (8) | 1 (8) | — | — | **—** |
| **Infections** |  |  |  |  |  |  |
| Bacterial | **7 (54)** | — | 3 (23) | 4 (31) | — | **4 (31)** |
| Viral | **7 (54)** | 1 (8) | 2 (15) | 4 (31) | — | **4 (31)** |
| **Constitutional** |  |  |  |  |  |  |
| Fatigue | **6 (46)** | 2 (15) | 4 (31) | — | — | **—** |
| Edema | **4 (31)** | 2 (15) | 2 (15) | — | — | **—** |
| Anorexia | **1 (8)** | 1 (8) | — | — | — | **—** |
| Dizziness | **1 (8)** | — | 1 (8) | — | — | **—** |
| Night sweats | **1 (8)** | 1 (8) | — | — | — | **—** |
| **Gastrointestinal** |  |  |  |  |  |  |
| Diarrhea | **5 (38)** | 3 (23) | 2 (15) | — | — | **—** |
| Constipation | **2 (15)** | 2 (15) | — | — | — | **—** |
| Nausea | **2 (15)** | 1 (8) | 1 (8) | — | — | **—** |
| Vomiting | **1 (8)** | 1 (8) | — | — | — | **—** |
| Pneumoperitoneum | **1 (8)** | — | 1 (8) | — | — | **—** |
| **Laboratory abnormality** |  |  |  |  |  |  |
| ALT increased | **3 (23)** | 1 (8) | 1 (8) | — | 1 (8) | **1 (8)** |
| AST increased | **2 (15)** | 1 (8) | — | — | 1 (8) | **1 (8)** |
| LDH increased | **2 (15)** | 2 (15) | — | — | — | **—** |
| Hypophosphatemia | **2 (15)** | — | 2 (15) | — | — | **—** |
| ALP increased | **1 (8)** | 1 (8) | — | — | — | **—** |
| Total bilirubin increased | **1 (8)** | 1 (8) | — | — | — | **—** |
| Hypogammaglobulinemia | **1 (8)** | 1 (8) | — | — | — | **—** |
| Hypokalemia | **1 (8)** | 1 (8) | — | — | — | **—** |
| Hyponatremia | **1 (8)** | 1 (8) | — | — | — | **—** |
| Hypernatremia | **1 (8)** | 1 (8) | — | — | — | **—** |
| **Musculoskeletal** |  |  |  |  |  |  |
| Bone pain | **3 (23)** | 2 (15) | 1 (8) | — | — | **—** |
| Pain in extremity | **2 (15)** | — | 2 (15) | — | — | **—** |
| Muscle spasms | **1 (8)** | 1 (8) | — | — | — | **—** |
| Myalgia | **1 (8)** | 1 (8) | — | — | — | **—** |
| **Dermatologic** |  |  |  |  |  |  |
| Rash | **3 (23)** | 2 (15) | — | 1 (8) | — | **1 (8)** |
| Dry skin | **1 (8)** | 1 (8) | — | — | — | **—** |
| Pruritus | **1 (8)** | 1 (8) | — | — | — | **—** |
| **Respiratory** |  |  |  |  |  |  |
| Cough | **2 (15)** | 1 (8) | 1 (8) | — | — | **—** |
| Nasal congestion | **2 (15)** | — | 2 (15) | — | — | **—** |
| Pneumonitis | **1 (8)** | — | 1 (8) | — | — | **—** |
| **Other** |  |  |  |  |  |  |
| Infusion-related reaction | **3 (23)** | — | 3 (23) | — | — | **—** |
| Chest pain | **2 (15)** | 2 (15) | — | — | — | **—** |
| Blurred vision | **2 (15)** | 1 (8) | 1 (8) | — | — | **—** |
| Peripheral neuropathy | **1 (8)** | — | 1 (8) | — | — | **—** |
| Hypotension | **1 (8)** | — | 1 (8) | — | — | **—** |
| Urinary tract pain | **1 (8)** | 1 (8) | — | — | — | **—** |
